# Resting easy with a sleep regulator

**DOI:** 10.7554/eLife.12093

**Published:** 2015-12-10

**Authors:** William J Giardino, Luis de Lecea

**Affiliations:** Department of Psychiatry and Behavioral Sciences, Stanford University, Stanford, United States; Department of Psychiatry and Behavioral Sciences, Stanford University, Stanford, United Statesllecea@stanford.edu

**Keywords:** hypocretin, orexin, zebrafish, sleep, transcriptome, kcnh4a, Zebrafish

## Abstract

Potassium ion channels in a subset of neurons in the brain of zebrafish may have a role in promoting sleep.

**Related research article** Yelin-Bekerman L, Elbaz I, Diber A, Dahary D, Gibbs-Bar L, Alon S, Lerer-Goldshtein T, Appelbaum L. 2015. Hypocretin neuron-specific transcriptome profiling identifies the sleep modulator Kcnh4a. *eLife* **4**:e08638. doi: 10.7554/eLife.08638

**Image** Mutations in the *Kcnh4a* gene reduce sleep in zebrafish.

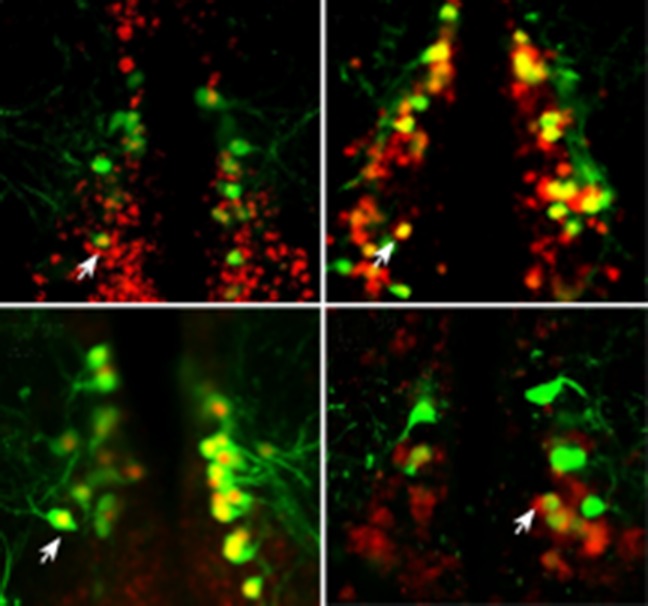

We are able to sleep as a result of a delicate balance between competing signals from complex circuits of neurons. In the hypothalamus, neurons that produce a neuropeptide called hypocretin (Hcrt; also known as orexin) control the transition between being asleep and being awake (Adamantidis et al., 2007; de Lecea, 2015). These neurons also coordinate waking up (arousal) in response to various stimuli (Giardino and de Lecea, 2014; Bonnavion et al., 2015). Thus, finding out how the activity of Hcrt neurons is regulated will be vital for understanding how these neurons maintain a healthy balance between the sleep and the awake states, and how this equilibrium is disturbed in individuals with sleep disorders. Now, in eLife, Lior Appelbaum and colleagues – including Laura Yelin-Bekerman as first author – report a new role for a potassium ion channel in the regulation of arousal (Yelin-Bekerman et al., 2015).

Yelin-Bekerman et al. – who are based at Bar-Ilan University, Toldot Genetics, the Weizmann Institute of Science and the Massachusetts Institute of Technology – studied zebrafish (*Danio rerio*), which display decreased movement patterns during the night that are characteristic of sleep. Moreover, they only have 16–20 Hcrt neurons, compared to more than 5000 cells in mammals. Using transgenic zebrafish that express enhanced green fluorescent protein only in their Hcrt neurons (*hcrt*-eGFP) (Faraco et al., 2006), Yelin-Bekerman et al. used a technique called fluorescence-assisted cell sorting to isolate the *hcrt*-eGFP cells and extract messenger RNA (Figure 1A). They then amplified the RNA material before carrying out whole-transcriptome sequencing to measure gene expression. Applying their most conservative criteria, Yelin-Bekerman et al. generated a list of 20 gene transcripts that are found at higher levels in Hcrt neurons than in other brain cells. These genes included *fam46a* and other markers of Hcrt neurons that have previously been reported in higher vertebrates (Dalal et al., 2013). With a more relaxed threshold, 212 transcripts met the criteria for being enriched in Hcrt neurons.

**Figure 1.**
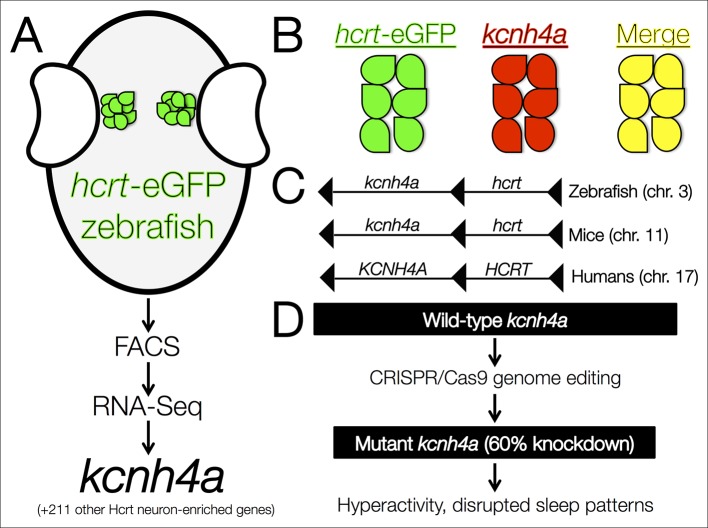
The *kcnh4a* gene is enriched in Hcrt neurons and promotes sleep in zebrafish. (****A****) Fluorescent-assisted cell sorting (FACS) of neurons from *hcrt*-eGFP zebrafish followed by all-mRNA sequencing (RNA-seq) identified 212 genes that are significantly enriched in Hcrt neurons, including the voltage-gated potassium channel *kcnh4a*. (****B****) In situ hybridization for *knch4a* confirmed its expression in 100% of *hcrt*-eGFP neurons. (****C****) Genomic analyses revealed that *kcnh4a* lies directly upstream of the *hcrt* gene, a phenomenon that is conserved from zebrafish to humans. (****D****) CRISPR/Cas9 genome editing produced a mutant zebrafish with a 60% reduction in *kcnh4a* levels, resulting in hyperactivity and disrupted patterns of nighttime sleep.

Using in situ hybridization, Yelin-Bekerman et al. then tested the degree to which the *hcrt*-eGFP and the 20 gene transcripts co-localize: they were able to confirm that several of the candidates are highly expressed within Hcrt neurons, and that others were actually expressed in nearby non-Hcrt neurons instead. Further analysis revealed that many of the genes contained conserved motifs for the binding of transcription factors, such as *pax4*, which is predicted to regulate as many as 44 genes in Hcrt neurons.

Of the transcripts that were verified as being enriched in Hcrt neurons, Yelin-Bekerman et al. focused on one called *kcnh4a*, which was the only one that was expressed in all *hcrt-*eGFP neurons in both zebrafish larvae and adults (Figure 1B). It encodes a potassium channel and further genetic analyses revealed that *kcnh4a* is located directly upstream of the *hcrt* gene in the zebrafish genome. In fact, these two genes are directly adjacent to each other throughout the animal kingdom, including in mice and humans (Figure 1C). The expression patterns and tight genetic linkage of the two genes suggests that well-preserved mechanisms govern their interactions at the protein level.

Pursuing this rationale, Yelin-Bekerman et al. examined whether the Kcnh4a protein is important for behaviors that are regulated by Hcrt neurons (Figure 1D). The authors used CRISPR/Cas9 genome editing to produce mutant zebrafish that had reduced levels of *kcnh4a* expression. Compared to wild-type zebrafish, these mutants showed significantly increased movement in both the light and dark periods of a 24-hour cycle. In further analyses in which sleep was defined as a period of inactivity that is longer than six seconds (Yokogawa et al., 2007), Yelin-Bekerman et al. reported decreased overall sleep time and shorter average bouts of sleep in *kcnh4a* mutants relative to control zebrafish, specifically during the dark periods.

An obvious next step is to determine whether Kcnh4a is also co-expressed with the Hcrt neuropeptide in mammals, and whether it plays a similar role in the sleep-wake transition. In vivo imaging with genetically-encoded calcium or voltage sensors in *kcnh4a* mutants will likely be useful in understanding the role of this potassium channel in the intrinsic properties of Hcrt neurons. Also, the unique properties of the zebrafish model make it possible to image the activity of single neurons and entire networks during 24-hour periods, and to monitor changes induced by sleep or wake-promoting drugs. In fact, Kchn4a is an intriguing potential new drug target, and may be highly relevant for therapeutic approaches to treat conditions like insomnia and narcolepsy.
